# Three-Dimensional Honeycomb-Like Porous Carbon with Both Interconnected Hierarchical Porosity and Nitrogen Self-Doping from Cotton Seed Husk for Supercapacitor Electrode

**DOI:** 10.3390/nano8060412

**Published:** 2018-06-08

**Authors:** Hui Chen, Gang Wang, Long Chen, Bin Dai, Feng Yu

**Affiliations:** 1 Key Laboratory for Green Processing of Chemical Engineering of Xinjiang Bingtuan, School of Chemistry and Chemical Engineering, Shihezi University, Shihezi 832003, China; huichen09@sina.com (H.C.); wanggang@shzu.edu.cn (G.W.); chenlong323@shzu.edu.cn (L.C.); db_tea@shzu.edu.cn (B.D.); 2 Key Laboratory of Materials-Oriented Chemical Engineering of Xinjiang Uygur Autonomous Region, Shihezi 832003, China; 3 Engineering Research Center of Materials-Oriented Chemical Engineering of Xinjiang Production and Construction Corps, Shihezi 832003, China

**Keywords:** nitrogen doping, hierarchical structure, porous carbon, three-dimensional architecture, cotton seed husk, supercapacitor, sustainable biomass

## Abstract

Hierarchical porous structures with surface nitrogen-doped porous carbon are current research topics of interest for high performance supercapacitor electrode materials. Herein, a three-dimensional (3D) honeycomb-like porous carbon with interconnected hierarchical porosity and nitrogen self-doping was synthesized by simple and cost-efficient one-step KOH activation from waste cottonseed husk (a-CSHs). The obtained a-CSHs possessed hierarchical micro-, meso-, and macro-pores, a high specific surface area of 1694.1 m^2^/g, 3D architecture, and abundant self N-doping. Owing to these distinct features, a-CSHs delivered high specific capacitances of 238 F/g and 200 F/g at current densities of 0.5 A/g and 20 A/g, respectively, in a 6 mol/L KOH electrolyte, demonstrating good capacitance retention of 84%. The assembled a-CSHs-based symmetric supercapacitor also displayed high specific capacitance of 52 F/g at 0.5 A/g, with an energy density of 10.4 Wh/Kg at 300 W/Kg, and 91% capacitance retention after 5000 cycles at 10 A/g.

## 1. Introduction

Supercapacitors are receiving extensive attention worldwide on account of their fast charge/discharge rates, higher power densities, and excellent cycling stabilities, which are superior to other energy storage devices [1,2,3]. The energy storage mechanisms of supercapacitors are mainly based on electrical double layer capacitance (EDLC), which occurs at the electrode/electrolyte interface [4,5], and pseudo-capacitors with reversible Faradic redox reaction [6]. Their relatively simple energy storage mechanisms and fast charge/discharge traits make supercapacitors the most promising energy storage devices [7,8,9]. However, the energy that is stored in a supercapacitor is lower than that in batteries. This has inspired research focused on increasing the supercapacitor’s energy density, while maintaining high power density [10].

Recently, studies have been conducted to produce porous carbon-based electrode materials, mainly focusing on porous structures [11,12]. In general, specific surface area (SSA) is the most important feature that influences the electrochemical performances of porous carbon materials [1,13,14]. High SSA benefits include good energy storage, especially for high rate performance. On the other hand, hierarchical pore size distribution accelerates the ion transfer from the electrolyte to electrode surface [15]. Typically, macro- and meso-pores provide space for electrolyte storage, thereby reducing the ion diffusion distance and ion diffusion resistance, which is conducive to better capacitive performance [16,17,18]. Additionally, three-dimensional (3D) frameworks are capable of supplying stable frame structures, inter-connected pore networks, and are further conducive to ion transfer [18,19]. However, in order to obtain above 3D hierarchical porous carbons (3D-HPCs), it is necessary to add a certain amount of hard templates or soft templates in most of the previously reported works [20,21,22]. Consequently, the synthesis of 3D-HPCs is limited by its complex, time-consuming, and costly processes.

Surface functional groups also play an important role in energy storage. Nitrogen doping is the most widely studied [23,24,25]. Generally, a proper amount of nitrogen doping promotes the electrochemical capacitance, by improving the wettability of the porous carbon electrode material, thus bringing about the properties of pseudo-capacitance [26,27,28,29]. Nitrogen-doped carbon is achieved by the pyrolysis of nitrogen-enriched polymer precursors and subsequent physicochemical activation, to obtain a nitrogen-doped carbon material. The process is expensive, time-consuming, and endangers our environment [30,31]. On the other hand, through the post-treatment of porous carbon with organic and inorganic nitrogen sources [32], urea [33,34] and ammonia [23,35], it is possible to obtain nitrogen-doped porous carbon. However, the resulting carbon material rarely has the previously mentioned 3D hierarchical structure. The traditional synthesis of 3D hierarchical nitrogen-doped porous carbon (3D-HNPCs) materials is limited to this complex, time-consuming, and costly process. Therefore, transforming sustainable raw materials into highly-performing 3D-HNPC materials, through a simple preparation method, is required.

In this work, we have successfully prepared a 3D hierarchical structure of nitrogen self-doped porous carbon from waste cottonseed husks (CSHs), for high performance supercapacitor electrode materials, using one-step KOH activation. After carbonization and activation, the obtained a-CSHs will have the following features. Firstly, it has a 3D architecture that is associated with hierarchical micro-, meso-, and macro-pores, a high specific surface area of 1694.1 m^2^/g, and a moderate pore volume of 0.87 cm^3^/g. Secondly, the obtained a-CSHs also contain a moderate nitrogen content of 2.56 atom %, which is as a result of the protein content in the raw materials. Thirdly, the synthesis process is also simple and convenient for large-scale industrial production. The formation of a 3D hierarchical structure is based on the fact that KOH acts both as hard template to form 3D structures and as an activator to produce affluent micropores on the surface of the carbon material. Finally, cotton is an important agricultural crop in China, with an annual production of 1.5 million tons [36]. The Xinjiang Production and Construction Corps occupies up to 70% of the total production. Cottonseed is mainly used to extract cottonseed oil, thereby generating a large amount of sustainable raw material. On the basis of the above advantages, a-CSHs provide high performed high supercapacitor performances in three- and two-electrode systems.

## 2. Materials and Methods

### 2.1. Sample Preparation

The preparation of nitrogen self-doped three-dimensional (3D) honeycomb-like porous carbon was synthesized by one-step KOH activation. The specific preparation steps were as follows: Cottonseed husk (CSH) was fully washed with deionized water to remove ash and other impurities, and then dried at 100 °C for 10 h. It was further crushed to form a powder and passed through a 200 mesh sieve for further use. Subsequently, the CSH powder was vigorously stirred with an aqueous KOH solution at a mass ratio of KOH/CSH powder = 1, and then dried at 80 °C. The mixture was then activated in a tube furnace under an Ar atmosphere at 600 °C, 700 °C, or 800 °C for 1 h with a heating rate of 5 °C/min. The obtained product was washed with 10% *v*/*v* HCl to remove the metal impurities, was washed with deionized water until the pH of the filtrate was 7.0, and was dried at 80 °C for 10 h. The obtained samples were denoted a-CSH-x, where x represented the activation temperature.

### 2.2. Material Characterization

Scanning electron microscopy (SEM) surveys were examined with a Hitachi SU8010 microscope (Tokyo, Japan). Transmission electron microscopy (TEM) and energy dispersive X-ray spectroscopy (EDS) were analyzed by a field emission Tecnai G2 F20 electron (Hillsboro, OR, USA) microscope. X-ray diffraction (XRD) measurements were carried out on a Bruker D8 Advance X-ray diffractometer with Cu-Kα radiation (Karlsruhe, Germany). Specific surface areas of the samples were calculated using the Brunauer-Emmett-Teller (BET) method (Micromeritics ASAP 2020 BET apparatus, Atlanta, GA, USA). The pore size distribution (PSD) curves were derived from the adsorption branch, using a nonlocal density functional theory (NLDFT) model assuming slit pore geometry. The surface chemical compositions were determined using an ESCALAB 250Xi (Thermo Fisher Scientific, USA) X-ray photoelectron spectroscope (XPS). The Raman spectra were collected on a LabRAM HR800 Laser Confocal Micro-Raman Spectroscope (Horiba Jobin Yvon, Franch) with a laser wavelength of 532 nm.

### 2.3. Electrochemical Measurements

The electrochemical properties of the as-prepared samples were tested on a CHI 760E working station with a 6 M KOH electrolyte. Cyclic voltammetry (CV) tests at different scanning rates and galvanostatic charge/discharge (GCD) curves under varying current densities were used to evaluate the electrochemical performances of the electrode materials. The working electrode was obtained by mixing carbon material (5 mg) with acetylene black (1 mg) and polytetrafluoroethylene (1 µL) in absolute ethanol (1 mL). The mixture was dispersed by ultrasound for 40 min and the ink-like dispersion that was obtained was transferred to nickel foam (1 cm × 1 cm) and then vacuum dried at 80 °C for 10 h. The nickel foam was further pressed on a tablet press at 20 MPa for 1 min and was immersed in 6 M KOH for further testing. The loaded mass of each electrode was 5 mg. For the three-electrode system, the Pt sheet and Saturated Calomel Electrode (SCE) were utilized as counter electrode and reference electrode, respectively. The specific capacitances of the samples were calculated through discharge curves following Equation (1), as follows:

(1) $$C=\frac{I\times \Delta t}{m\times \Delta V}$$

where *C* (F/g) is the specific capacitance, *I* (A) is the charge/discharge current, Δ*t* (s) is the discharging time, *m* (g) is the mass of the working electrode, and Δ*V* (v) is the voltage window of the charge/discharge process. 

The electrochemical properties of a-CSH-700 were measured with the two electrode system. Two symmetrical electrodes were separated by a cellulose membrane in a 6 M KOH electrolyte and were assembled in a CR2032 stainless-steel coin cell. The specific capacitance was calculated from the discharge process, according to Equation (1). The energy density and power density of symmetric supercapacitor systems were further calculated by Equations (2) and (3).

(2) $$E=\frac{1}{2}C_{t}\Delta V^{2}\times \frac{1}{3.6}$$

(3) $$P=\frac{E}{\Delta t}\times 3600$$

where *E* (Wh/kg), *P* (W/kg), *C*_t_ (F/g), Δ*V* (v), and Δ*t* (h) are the specific energy density, specific power density, specific capacitance, and voltage window, respectively, of the symmetrical supercapacitor system.

## 3. Results

**Figure 1:**
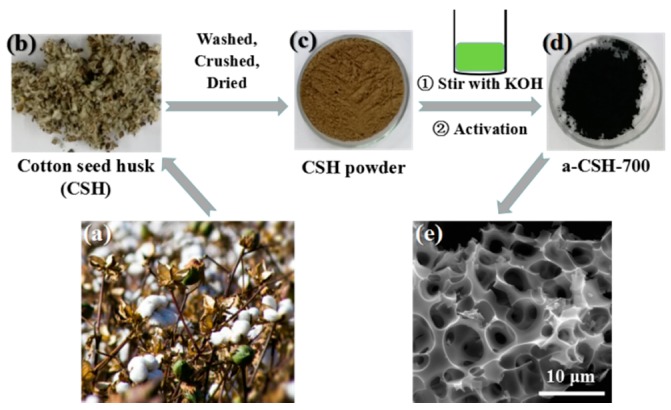
Schematic diagrams for the fabrication of a-CSH-700 and the corresponding scanning electron microscopy (SEM) image. (**a**) Images of cotton. (**b**–**d**) Schematic of the synthesis of a-CSH-700 derived from cottonseed husk, and (**e**) the corresponding SEM images of a-CSH-700.

Nitrogen self-doped 3D honeycomb-like porous carbon was prepared through one-step activation from waste cottonseed husk (CSH). Cotton seeds were generally collected for the preparation of cottonseed oil; large amounts of cotton seed husks were not effectively used and were abandoned. Therefore, we recycled them to prepare high-performance biomass-derived electrode materials (Figure 1a,b). The material preparation process is shown in Figure 1b–d. Pretreated CSH powder (Figure 1c) was directly stirred with aqueous KOH solution and dried for carbonization and activation. The temperature for activation was adjusted from 600 °C to 800 °C, and the carbon that was obtained was washed and dried. The entire preparation process was cost-efficient, simple, and easily achieved the industrialized requirements.

**Figure 2:**
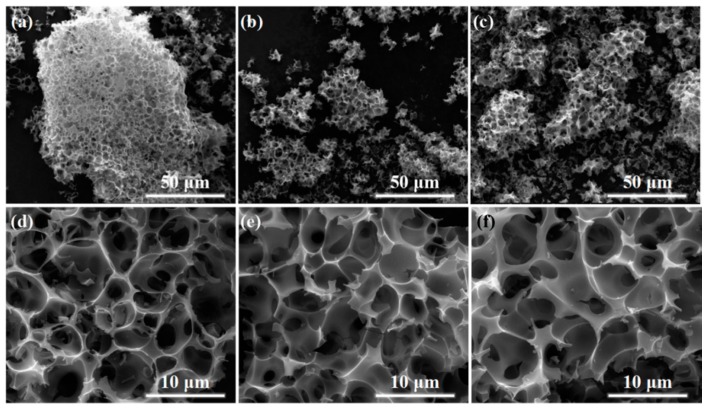
SEM images of (**a**,**d**) a-CSH-600, (**b**,**e**) a-CSH-700, and (**c**,**f**) a-CSH-800.

**Figure 3:**
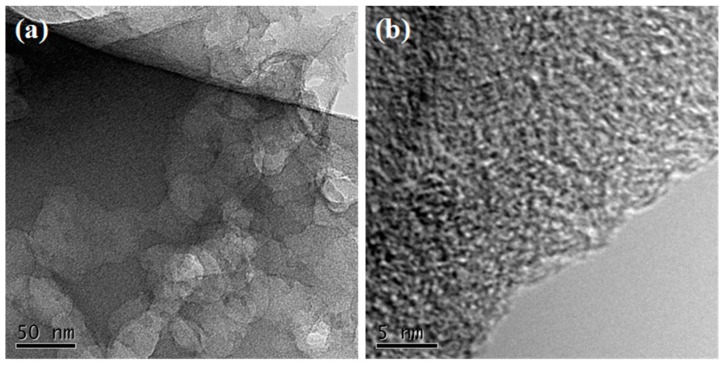
Transmission electron microscopy (TEM) image (**a**) and high-resolution TEM (HRTEM) image (**b**) of a-CSH-700.

Scanning electron microscopy (SEM) images of a-CSH-600, 700, and 800 are shown in Figure 2a–f. The micromorphologies of the obtained samples showed typical 3D inter-connected honeycomb-like microstructures at different pyrolysis temperatures. The chemical composition of the waste cottonseed husk had a certain degree of degradation after stirring and evaporation with an aqueous KOH solution. Subsequently, the following chemical reaction of CSH and KOH during carbonization and activation processes occurred: 6KOH + C → 2K + 3H_2_ + 2K_2_CO_3_, followed by the decomposition of K_2_CO_3_, and the simultaneous generation of 3D pore structures and graphite sheet-like layer structures [8,37,38]. The lateral size of the 3D porous carbon varied in the range 400 nm to 4 μm (Figure 1d–f). With the increasing pyrolysis temperature, the characteristics of the 3D structure were slightly damaged, which was mainly because the higher temperature was bad for obtaining the 3D structure. The 3D linked carbon skeleton caused the obtained carbon material to exhibit a higher specific surface area. Moreover, this unique 3D structure generated abundant interconnected pore structure that allowed the electrolyte to be stored therein, reducing the distance that the electrolyte travelled on the surface of the electrode material. At the same time, abundant micropores and mesoporous structures existed on the surface of the carbon material (Figure 3a,b). With the help of these multi-level pore structures, it was easy to obtain a high energy storage performance.

**Figure 4:**
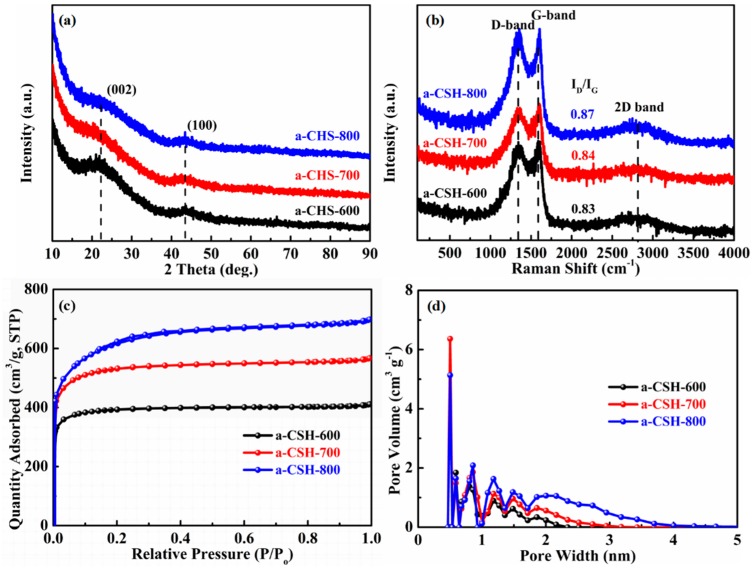
Powder X-ray diffraction (XRD) patterns (**a**) and Raman spectra (**b**) of a-CSHs samples. (**c**) Nitrogen adsorption-desorption isotherms and (**d**) pore size distributions of a-CSHs.

The powder X-ray diffraction (XRD) patterns of as-prepared a-CSHs samples are shown in Figure 4a. There were two distinct peaks at around 2θ = 22.1° and 43.5°, which were ascribed to the (002) and (100) reflections of the amorphous graphitic carbon structure. The high intensity values at the low angles indicated high specific surface areas of carbon materials. The obvious peak at 43.5° revealed a higher degree of interlayer condensation in a-CSHs, which also significantly increased the electrical conductivity. Raman spectroscopy was further used to characterize the a-CSHs samples. As shown in Figure 4b, there were three distinct peaks at 1343 cm^−1^ (D band), 1590 cm^−1^ (G band), and 2800 cm^−1^ (2D band). The D band represented the degree of the defects and disordered sp^3^ carbon atoms in the sample, and the G band was in line with graphite sp^2^ hybridized carbon atoms in the sample. The presence of a 2D peak indicated that there existed an ordered graphite-like structure in the a-CSHs. The intensity ratio of D band to G band (I_D_/I_G_) represented the disorder degree of the samples [39,40]. The I_D_/I_G_ ratios of a-CSH-600, a-CSH-700, and a-CSH-800 were 0.83, 0.84, and 0.87, respectively. We confirmed that the chemical reaction became deeper with rise in activation temperature, which promoted the defects and disordered structures in a-CSH-700 and a-CSH-800.

The nitrogen adsorption/desorption measurements were further used to examine the pore properties of a-CSHs. The nitrogen adsorption-desorption isotherm and pore size distribution curve of a-CSHs are shown in Figure 4c,d, respectively. It could be seen that all of the a-CSHs samples displayed type I isotherms [41]. With the increase in pyrolysis temperature, the corresponding quantity adsorbed value also increased [42]. This suggested that the specific surface area increased with an increase in the activation temperature. Figure 4d displays the pore size distribution isotherms. The dominant pore size distribution was located in the micropores (0.5–2 nm), and a part in mesopores (2–4 nm). a-CSH-600, a-CSH-700, and a-CSH-800 exhibited hierarchical porous structures, abundant micropores, and profuse mesopores, respectively, which were consistent with the results of the SEM images. Table 1 also summarizes the information on the specific BET surface areas and pore sizes of all of the a-CSHs samples. The specific surface areas of a-CSH-600, a-CSH-700, and a-CSH-800 were determined to be 1257.8, 1694.1, and 2063.0 m^2^/g, respectively, while the pore volumes were 0.64, 0.87, and 1.07 cm^3^/g, respectively. This showed that the temperature of activation was a dominating factor for the development of pore structure. 

**Figure 5:**
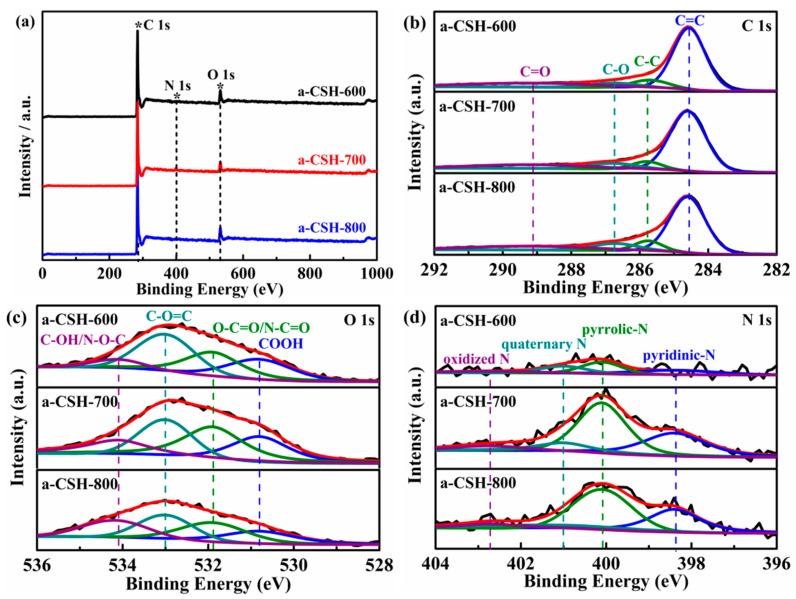
XPS images of the a-CSH-600, a-CSH-700, and a-CSH-800 (**a**). High-resolution C 1s (**b**), O 1s (**c**), and N 1s (**d**) of the a-CSH-600, a-CSH-700, and a-CSH-800.

**Figure 6:**
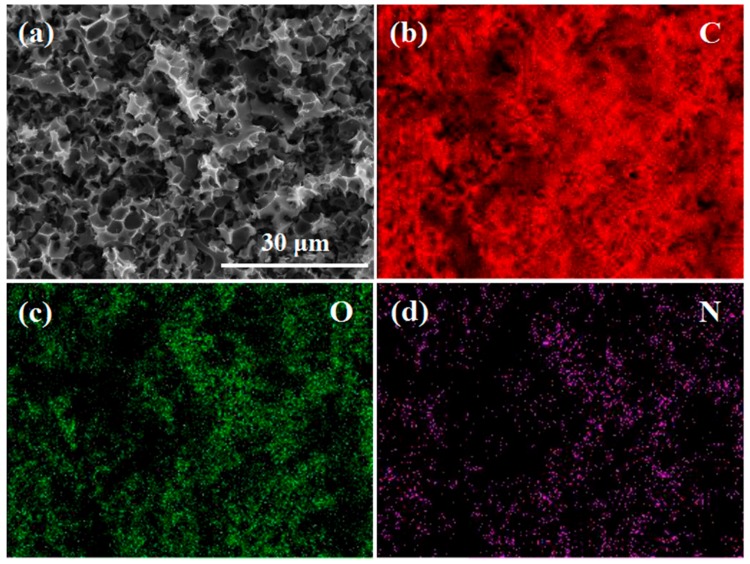
(**a**) SEM image of a-CSH-700, and elemental mapping images of (**b**) C, (**c**) O, and (**d**) N.

The chemical compositions and surface functional groups of as-prepared a-CSH-600, a-CSH-700, and a-CSH-800 were further characterized by XPS measurements. As shown in the survey spectra (Figure 5a), there existed three distinct peak signals corresponding to the C 1s peak near 286 eV, the N 1s peak near 400 eV, and the O 1s peak near 534 eV. Table 2 lists the statistical results of the corresponding elemental contents of the as-prepared carbon materials. The carbon content decreased and the associated oxygen content increased with rise in activation temperature. The nitrogen content of a-CSHs ranged from 1.51 to 2.56 atom %, the main reason being the complex chemical processes and structure features of these groups at a higher temperature. Figure 6 shows the element mapping images of as-obtained a-CSH-700. The nitrogen was uniformly distributed on the surface of the carbon, and the nitrogen content was 2.62 atom %. 

Figure 5b–d displays high resolution C 1s, O 1s, and N 1s spectra of the as-prepared carbon materials. The high resolution C 1s spectrum (Figure 5b) of a-CSHs was resolved into four individual peaks at 284.6, 285.7, 286.7, and 289.2 eV, corresponding to C=C, C–C, C–O, and C=O, respectively [43,44]. The high-resolution O 1s spectrum at 530.8, 531.9, 533, and 534.1 eV were associated with COOH, O–C=O/N–C=O, C–O=C, and C–OH/N–O–C, respectively [45,46]. The high-resolution N 1s spectrum was composed of pyridinic-N (398.4 eV), pyrolic-N (400.1 eV), quaternary-N (401 eV), and oxidized-N (402.7 eV) [47,48,49]. These nitrogen and oxygen functional groups enhanced the surface wettabilities of the a-CSHs, and thereby promoting the specific capacitances of the as-prepared carbon materials [8,50,51].

The general standards for designing high performance supercapacitor electrodes were associated with high specific capacitance, good rate capability, and long cycle stability. According to the above conclusions, the a-CSHs derived from one-step synthesis possessed many advantages for supercapacitor electrode materials. The a-CSHs had superior specific surface area for forming ideal electrochemical double layers. The interconnected micropores and mesopores could increase ion transport and the abundant surface nitrogen functional groups improved the wettability and promoted electrical conductivity. The 3D structure provided space for electrolyte storage. These characteristics endowed a-CSHs with a high supercapacitor performance.

**Figure 7:**
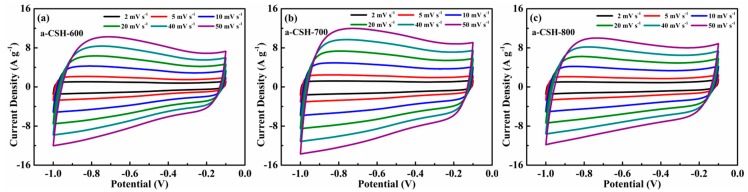
Electrochemical performance characteristics of a-CSHs measured in a three-electrode system in a 6 M KOH electrolyte: the cyclic voltammetry (CV) curves of (**a**) a-CSH-600, (**b**) a-CSH-700, and (**c**) a-CSH-800 at different scan rates.

**Figure 8:**
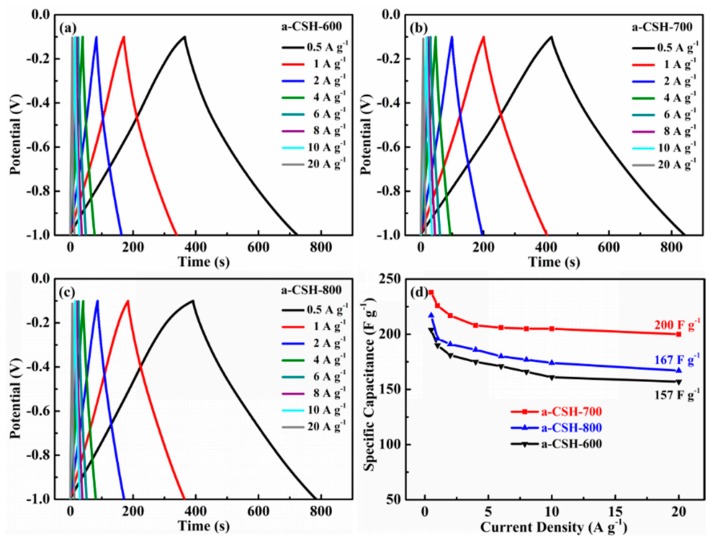
Electrochemical performance characteristics of a-CSHs measured in a three-electrode system in a 6 M KOH electrolyte: galvanostatic charge/discharge (GCD) curves of (**a**) a-CSH-600, (**b**) a-CSH-700, and (**c**) a-CSH-800 at different scan rates. (**d**) Specific capacitances at different current densities.

The electrochemical performances of the obtained a-CSHs were tested by a three-electrode system in a 6 M KOH aqueous electrolyte and the CV curves are shown in Figure 7. The CV curves of a-CSH-600 (Figure 7a), a-CSH-700 (Figure 7b), and a-CSH-800 (Figure 7c) showed typical quasi-rectangular shapes at scan rates from 5 mV/s to 50 mV/s. This suggested that all of the samples displayed ideal electrochemical double layer capacitances. The CV curves of a-CSH-700 had the largest current responses and areas, suggesting the highest capacitance. Furthermore, galvanostatic charge/discharge (GCD) was further used to assess the electrochemical performance, which is shown in Figure 8. The GCD curves of a-CSH-600 (Figure 8a), a-CSH-700 (Figure 8b), and a-CSH-800 (Figure 8c) showed typical quasi-linear shapes at current densities from 0.5 A/g to 20 A/g. It also suggested that the as-obtained carbon samples possessed good electrochemical performances. Figure 8d summarizes the gravimetric specific capacitances of a-CSHs, calculated from galvanostatic charge/discharge curves at a current density ranging from 0.5 to 20 A/g. The specific capacitance value of the a-CSH-700 sample was as high as 238 F/g at a current density of 0.5 A/g, which was higher than those of a-CSH-800 (217 F/g) and a-CSH-600 (204 F/g). The capacitance was also as high as 200 F/g for a-CSH-700 with an excellent capacitance retention of 92%, indicating its outstanding rate capability. For a better comparison, Table 3 lists the specific capacitances of other biomass-derived porous carbons that were reported in the recent literature. Although the electrode of a-CSH-700 exhibited the best electrochemical performance, its specific area and surface functional groups were not the highest among all of the samples, meaning that there were synergistic effects.

**Figure 9:**
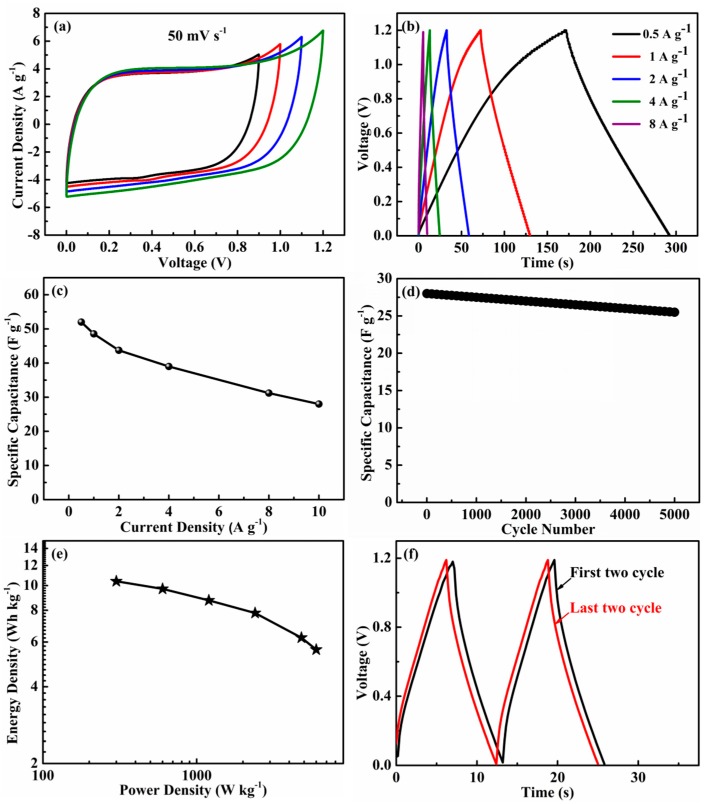
Electrochemical measurements of an as-assembled a-CSH-700//a-CSH-700 symmetric supercapacitor in a 6 M KOH electrolyte: (**a**) CV curves of the cell operated in different voltage windows at scan rate 50 mV/s; (**b**) galvanostatic charge/discharge curves of the cell at various current densities; and (**c**) specific capacitances for the supercapacitor at different current densities. (**d**) Cycling stabilities of the devices at a current density of 10 A/g. (**e**) Ragone plot. (**f**) First two cycle charge-discharge and last two charge-dischage plots.

The capacitive performance of a-CSH-700 was further evaluated by assembling it in a symmetric two-electrode cell in 6 M KOH. Figure 9a shows the CV curves that were tested in different potential windows. It could be seen that there no obvious promotion of anodic current when the operating voltage was 1.2 V. The GCD curves (Figure 9b) had a symmetric triangular shape as the current density increased from 0.5 A/g to 10 A/g, and there were no evident IR drops. The testing results also showed that it had a good capacitive performance. The specific capacitance for the entire electrochemical supercapacitor was estimated to be 52 F/g at 0.5 A/g with an energy density of 10.4 Wh/kg and power density 300 W/kg (Figure 9c,e). Figure 9d shows the cycling stability of the device at a higher current density of 10 A/g. It could be seen that the charge/discharge curves of the last two cycles were the same as the first two cycles (Figure 9f). Capacitance retention was also as high as 91% after 5000 cycles. These results revealed that the synthesized a-CSH-700 sample was a superior electrode material for high power and cost-effective supercapacitors.

## 4. Conclusions

Nitrogen self-doped 3D honeycomb-like porous carbon was successfully prepared via one-step KOH activation (a-CSHs). When the carbonization temperature was adjusted, the obtained carbon materials showed high specific surface areas (1257.8–2063.0 m^2^/g), abundant nitrogen contents (1.51–2.56 atom %), and hierarchical pore structures. The a-CSH-700 based electrode delivered a high specific capacitance of 238 F/g and 200 F/g at current densities of 0.5 A/g and 20 A/g, respectively, demonstrating a good capacitance retention of 84%. Moreover, the assembled a-CSH-700 based symmetric supercapacitor also displayed a high specific capacitance of 52 F/g at 0.5 A/g, with an energy density of 10.4 Wh/kg at 300 W/kg, and 91% capacitance retention after 5000 cycles at 10 A/g. This facile and cost-effective method for the preparation of 3D honeycomb-like porous carbon material from waste cottonseed husk was also renewable and easy for industrial production.

## Figures and Tables

**Table 1:** Pore characteristics of the a-CSHs samples.

Samples	S_BET_^a^ (m^2^/g)	S_mi_^b^ (m^2^/g)	V_total_^c^ (cm^3^/g)	V_mid_^b^ (m^2^/g)	D_aver_^d^ (nm)
a-CSH-600	1257.8	1051.0	0.64	0.51	3.95
a-CSH-700	1694.1	1253.5	0.87	0.63	3.76
a-CSH-800	2063.0	1080.0	1.07	0.52	2.60

^a^ Total surface area calculated using the Brunauer-Emmett-Teller (BET) method; ^b^ Micropore surface area and volume calculated from the t-plot method; ^c^ Total pore volume calculated at P/P_o_ = 0.99; ^d^ Average pore diameter calculated from the (Barrett-Joyner-Halenda) BJH desorption.

**Table 2:** Elemental contents of a-CSHs samples from X-ray photoelectron spectroscope (XPS) and EDS.

Samples	Composition (from XPS)			(from EDX)
	C (atom %)	O (atom %)	N (atom %)	N (atom %)
a-CSH-600	90.03	8.46	1.51	1.87
a-CSH-700	90.11	7.33	2.56	2.62
a-CSH-800	91.19	6.37	2.44	2.86

**Table 3:** Electrochemical performance of biomass derived porous carbons.

Materials	S_BET_ (m^2^/g)	C_m_ (F/g)	Current Density	Electrolyte	Ref.
Broad beans	655.4	129	10 A/g	6 M KOH	[52]
Banana peel	1357.6	155	2.5 A/g	6 M KOH	[44]
Potato waste	1052	192	10 A/g	2 M KOH	[53]
Banana peel	1650	182	10 A/g	6 M KOH	[54]
Sugarcane bagasse	1939.6	175	20 A/g	1 M H_2_SO_4_	[55]
Pomelo	974.6	176.4	20 A/g	2 M KOH	[56]
Chitin	1600	196.2	20 A/g	6 M KOH	[57]
Cotton seed husk	1694.1	200	20 A/g	6 M KOH	This work
